# Bibliology; or Analytical Reviews of Medical Publications, Interwoven with Original Observations, and Practical Researches

**Published:** 1819-04-01

**Authors:** 


### SECOND DEPARTMENT.

### BIBLIOLOGY;

OR,

ANALYTICAL REVIEWS OF MEDICAL PUBLICATIONS,

*Interwoven with*

ORIGINAL OBSERVATIONS, AND PRACTICAL RESEARCHES.

Nec tibi quid liceat sed quid fecisse decebit
Occurat, mentemque domat respectus honesti.

CLAUD.

### Medicine.

### I.

*A Popular Treatise on the Remedies to be employed in Cases of poisoning and apparent Death; including the Means of detecting Poisons, of distinguishing real from apparent Death, and of ascertaining the Adulteration of Wines.* By M. P. ORFILA, &c. &c. &c. 8vo. pp. 170. London, 1818. Translated from the French, by W. PRICE, M. D.

THE work of death is so quickly performed by the stronger poisons, in the hands of ignorance or villainy, and the consternation is so great on the part of the bye-standers, and even medical men themselves, that a plain and concise code of instructions for the guidance of the practitioner, on these emergencies, is a great desideratum.

We have often thought that a well-constructed chart, or large sheet of paper, where the various poisons, their tests—the symptoms produced by their introduction into the body, and their proper antidotes, were clearly arranged, would be the means of saving many lives annually. We had thoughts of forming such a chart; but our other avocations will not now permit us. Mean time, we shall here endeavour to lay before the professional reader a Toxicological Epitome, being a partial analysis of the volume before us, which may prove of essential service in the moment of exigency, when every *moment* is of the utmost value.

### CLASS I. MINERAL POISONS.

### SECTION 1.—The ACIDS; viz. *The Sulphuric, Nitric, Muriatic, &c.*

I. *Tests.* All the acids redden the blue tincture of turnsole. The sulphuric has no odour. Concentrated nitric acid is white; poured upon copper, it bubbles and raises orange-yellow coloured vapours. Concentrated muriatic acid gives out white vapours.

II. *Symptoms, when taken into the Stomach.* Disagreeable acid, burning taste; heat in the throat and stomach; acute pain in the throat descending to the bowels; insupportable fœtor of the breath; inclination to vomit; vomiting of different-coloured matters, sometimes reddened with blood, and reddening tincture of litmus; hiccup; constipation, or frequent bloody stools; colic, or rather acute pain in the belly; difficulty of respiration; anguish; frequent irregular pulse; burning thirst; pain augmented by drinking. Convulsions, and various other distressing symptoms terminate the scene.

III. *Antidotes.* Calcined magnesia. The patient should take immediately a mixture of magnesia and water, in the proportion of an ounce to a pint. A glass of this should be given every two minutes, in order to produce vomiting. While the magnesia is procuring, common water, or linseed decoction should be drank. When magnesia cannot be procured, half an ounce of soap dissolved in a pint of water, should be given. Where these cannot be procured, prepared chalk may be given. Glysters prepared with the same substances should also be given. Potassa and soda are too irritating.

IV. *Treatment.* Certain of having neutralized all the acid which has not acted, our next object is to obviate the inflammation induced. Leeches and fomentations should be applied to the abdomen; and if prompt relief be not obtained by these and the warm bath, blood is to be taken from the arm, in greater or less quantities, according to the urgency of the symptoms. All food is to be prohibited—diluents are to be taken, and laxative glysters thrown up. If the patient cannot swallow, from inflammation of the throat, leeches are to be applied there. The cramps and spasmodic affections will disappear with the inflammation which produced them. A return to diet during convalescence must be extremely gradual.

Section ii. Concentrated Alkalies, *as pure Potash, Subcarbonate of Potash, Soda, Liquid Ammonia, Lime, &c.*

i. *Tests.* The alkalies dissolved in water, colour the syrup of violets green.

ii. *Symptoms, when taken into the Stomach.* These are nearly the same as those where the acids are taken. The taste of these poisons, however, is acrid, caustic, and urinous; and the matter vomited changes to green the syrup of violets. The concentrated volatile alkali soon occasions dreadful convulsions—it is even dangerous to apply it long to the nostrils of a person who has fainted. The vapour evaporates, and inflames the throat and lungs, as has recently been exemplified fatally.

iii. *Antidotes.* Vinegar and lemon-juice. Two table spoonfuls of vinegar or lemon-juice, should be put to a glass of water, and several glasses thus acidulated should be administered with the greatest possible promptitude. Where these are not at hand, plain water should be taken in large quantities, carefully avoiding emetic tartar, ipecacuanha, or other irritating substances. The sequelæ or inflammatory effects must be obviated, as where the acids have been swallowed.

Section iii. *Corrosive Sublimate, Arsenic, Verdigris, Blue Vitriol, Antimony.*

Before speaking of these individually, some general remarks may be made on them collectively.

The taste of these substances is acrid and metallic. The patient complains of a constriction of the throat; pain in the back part of the mouth, in the stomach, and in the bowels; vomiting of various matters, which never colour the syrup of violets green; and but faintly redden the blue tincture of turnsole; constipation or diarrhœa. To these are soon superadded, fœtid eructations, hiccups, difficulty of breathing. The pulse becomes accelerated, small, and hard—sometimes intermitting; thirst unquenchable; difficulty of voiding urine; cramps; delirium; convulsions—death.

i. *Corrosive Sublimate,* [Oxymurias Hydrargyri]. This medicine cannot be taken with impunity, even in doses of one or two grains. Placed upon wounds, cancers, ulcers, &c. it acts as a violent poison, and death ensues in ten, fifteen, twenty, or thirty hours. Hence the danger of its external application.

*Tests.* All the mercurial preparations, heated to redness in a glass tube with potash, are decomposed, the quicksilver being set at liberty and volatilized. The corrosive sublimate is white, soluble in water, and is precipitated *yellow* by potash, and *white* by ammonia.

*Antidotes.* Whites of eggs beat up with water—milk. When an individual is poisoned by a mercurial preparation introduced into the stomach, or applied externally, the whites of twelve or fifteen eggs [the yolks may be included without inconvenience] should be mixed up with two pints of cold water, and a glass of this drink given every two minutes, in order to promote vomiting. If the above number cannot be procured, then as many as possible. When eggs are not at hand, milk should be given in abundance; and where neither can be got, then gum water; infusion of linseed, of marshmallows; sugar and water; or, if all are absent, pure water itself should be administered. The after-treatment of inflammatory sequelæ is the same as mentioned under acids.

II. *Arsenic.—Tests.* White arsenic is under the form of a white powder, like sugar, but is heavier; volatilises, diffusing an odour of garlic when thrown upon burning coals. It is soluble in water; becomes of a very fine green when put into blue ammoniacal sulphate of copper. The arsenic acid is white, diffuses an odour of garlic when thrown upon burning coals. Dissolves very readily in water, and passes to a very clear blue when put into ammoniacal sulphate of copper.

*Symptoms when taken into the System.* The external application of arsenic to cancers, &c. is very dangerous, since experience has proved that it may cause all the symptoms of poisoning, and occasion death in twenty-four or forty-eight hours. Swallowed, even in very small doses, arsenic is a strong poison. It does not kill, as is vulgarly supposed, by burning the stomach and intestines, but by being absorbed, and thus destroying the vital properties of the heart—very often this organ is inflamed and ulcerated by the arsenic.

*Antidotes.* Sugar and water; decoction of the roots of linseed or mallows. By these means the stomach is filled, vomiting is induced, and the poison is necessarily ejected. Equal parts also of lime water and sugar and water may be given.

The after-treatment the same as where acids are swallowed.

III. *Preparations of Copper.* The more active preparations of copper are poisonous, even when taken in small doses. They may, however, be applied to wounds without danger.

*Tests.* The salts of copper dissolved in water are, in in general, of a blue green colour. The Prussiate of potash gives a reddish brown precipitate, metallic iron and phosphorus instantly separate the copper. The artificial verdigris is not entirely soluble in cold water, it affords a blue liquor and a blackish brown powder. Exposed to a red heat in a crucible, it is decomposed, and yields metallic copper.

*Antidotes.* Albumen; for instance, whites of eggs.

*Treatment.* A person poisoned by verdigris, or by any other copper salt, ought to be treated as directed in treating of corrosive sublimate.

### Section IV.—Antimonial Preparations.

The effects of these are very similar to those where acids are swallowed, excepting that they occasion more abundant and obstinate vomiting; very copious stools; difficult respiration; and often such a constriction of the throat, that the patient cannot swallow. Afterwards, they produce painful cramps, a sort of intoxication, and prostration of strength.

*Tests.* All the preparations of antimony, when exposed to a red heat, in a crucible, with potash and charcoal, yield metallic antimony, which is easily known; 1st. by its bluish white colour; 2d. by the property which it has, when heated with nitric acid, of giving a white powder, soluble in hydro-chloric acid:—this solution gives an orange precipitate with hydro-sulphuric acid, and a white one with water. Tartar emetic is white: thrown upon burning coals, it becomes black, and affords metallic antimony. It is soluble in water; the solution is not made turbid by distilled water; it is precipitated of an orange colour by the hydro-sulphuric acid—of a greyish white by the infusion of galls.

*Treatment.* If the individual has not begun to vomit, and yet presents the symptoms of being poisoned, several glasses of *sugar and water* should be given. If they do not produce vomiting, four or five gall-nuts broken small, or two ounces of bark grossly powdered, should be boiled in two pints of water, for ten minutes: for the want of these substances, oak or willow bark may be used; several glasses of this drink should be given.

If, on being called to the patient, in such cases, there have been abundant vomitings, and there are cramps and pains of the stomach, &c. the vomiting must be promoted by the administration of *sugar and water*, in repeated draughts. If, notwithstanding these means, the vomitings and pains continue or augment, a grain of the extract of opium should be given, dissolved in a glass of sugar and water, and repeated three times, at intervals of a quarter of an hour.

If the symptoms should still continue or augment, twelve or fifteen leeches should be applied upon the region of the stomach; and if the constriction of the throat should prevent swallowing, the same application should be made to the neck.

### Section v.—Preparations of Silver.

The nitrate of silver, [lunar caustic] though useful when employed in epilepsy, and some other diseases, is yet poisonous when swallowed. Applied externally, it is quite innocent. It may be known by the following characters:—1st. On exposing it to a red heat, the silver will be reduced. 2d. On dissolving it in water, the liquid will give a white precipitate with solution of common salt; a yellow with the phosphate of soda; and a red one with chromate of potash.

*Treatment. Common salt* is the best antidote against nitrate of silver. A person poisoned with this substance therefore, should drink several glasses of salt and water—a table spoonful to two pints of water. Vomiting will follow, and the symptoms will diminish. If, by chance they continue, recourse should be had to leeches, emollient drinks, fomentations, and all the measures indicated in the treatment of poisoning by acids.

### Section vi.—Nitre [*Saltpetre, Nitrate of Potash*].

*Distinctive Characters.* Thrown upon burning coals, nitre crackles, and gives a beautiful white flame; on the contrary, Glauber's salt [sulph. sodæ with which it is principally confounded] melts, puffs up, and becomes opake. Reduced to a fine powder and mixed with the oil of vitriol [sulphuric acid] nitre gives out white vapours, which is not the case with Glauber's salt.

*Effects when swallowed.* It gives rise to obstinate and bloody vomitings, to an active inflammation of the stomach, and consequently to the symptoms attending this inflammation. It also affects the nervous system, often occasioning a sort of intoxication, palsy of the limbs, convulsions, and other nervous diseases.

*Treatment.* The same as for arsenic [Sect. III—II.] except that lime water is not to be given. The after-treatment the same as where acids are taken [Sect. I. IV.]

SECT. VII. *Muriate of Ammonia* [Sal ammoniac] occasions vomitings, convulsive movements, a general stiffness—pains in the belly, alteration of the features, and death.

*Treatment.* Vomiting should be immediately induced by swallowing several glasses of water, or of sugar and water; by introducing the fingers into the mouth, and by tickling the throat with a feather. The nervous affections should afterwards be calmed by anodynes, and inflammation counteracted by the usual means.

*Distinctive Characters.* Exposed to heat, sal-ammoniac volatilises, producing a white vapour; triturated with quick lime, it diffuses the odour of volatile alkali or hartshorn; dissolved in water and mixed with the nitrate of silver, it occasions a very heavy white precipitate.

SECTION VIII.—PREPARATIONS OF BARYTES. [*Protoxide of Barytes, or terra ponderosa—Hydro-chlorate of Barytes, or Muriate of Barytes.*]

These preparations are extremely poisonous when taken into the stomach, or applied to wounds, occasioning vomitings, convulsions, palsy of the limbs, pains in the belly, hiccups, alteration of the countenance, and death.

*Characters.* All the soluble preparations of barytes, mixed with well water, or with a solution of Glauber's or Epsom salts, give a white precipitate, insoluble in water and in nitric acid.

*Treatment.* A weak solution of sulphate of soda, or sulphate of magnesia should immediately be swallowed—for example, half an ounce of either of these salts dissolved in a pint of water. If these are not at hand, well water must be used. When this treatment shall have promoted vomiting, decomposed the poison, and calmed the principal symptoms, then some sugar and water should be given, or any other emollient drink. The after-treatment will be easily understood.

### Section ix.—*Lytta. Cantharides*

*Symptoms when swallowed.* Nauseous odour on the breath; acrid and very disagreeable taste in the mouth; burning heat in the throat, stomach, and other parts of the abdomen; inclination to vomit; frequent vomitings, often mixed with blood; copious, and more or less, bloody stools; excruciating pain in the belly, particularly towards the stomach; obstinate and painful priapism; heat in the bladder; great difficulty in voiding urine; sometimes suppression of urine; pulse frequent and hard; occasional inability to swallow; at last frightful convulsions, delirium, and death.

*Treatment.* The patient should drink a large glass of sweet oil to produce vomiting; or, in the want of this substance, several glasses of water, or what is better, sugar and water, milk, decoction of mallows, or linseed tea. One or other of these emollient liquids should be injected into the bladder to prevent inflammation there. The symptoms of internal inflammation must be combated on general principles.

### Section x.—*Glass, or Enamel.*

These, when finely powdered, may be swallowed with impunity; but if they are in pointed fragments, they produce the same inconvenience as other sharp bodies, by lacerating and inflaming the mucous membrane of the stomach. In such cases, and where there are pains in the stomach, with heat, and other symptoms, the person should eat large quantities of beans, potatoes, cabbage, bread, or any other usual aliment. By this means the stomach will be filled, and the glass involved. Two or three grains of tartarized antimony should then be given, dissolved in a glass of water. Vomiting will thus be excited and the glass evacuated. Milk should afterwards be given, emollient glisters, fomentations, and warm bath. If gastric inflammation succeed, then it must be treated on general principles.

### Section xi.—Preparations of Lead.—*Superacetate of Lead—Plumbi Superacetatis—Cerusse—Litharge—Red Lead—Wine sweetened with Lead, &c.*

When a considerable dose of sugar of lead, or any other preparation of this kind, soluble in water, has been swallowed, the patient experiences a sugary, astringent, metallic, disagreeable taste; a constriction of the throat, pain in the region of the stomach, inclination to vomit, obstinate, painful, and often bloody vomitings; in short, all the symptoms which result from an inflammation of the stomach, or from taking corrosive sublimate. When taken gradually in small quantities, and long continued, it produces a chronic disease, resembling the Painter's Colic, but which, in certain circumstances, is true palsy.

*Tests.* A salt of lead in solution, or water containing the metal, may be detected by pouring into it—1st, some sulphuric acid, which will throw down a white precipitate; 2d, some sulphuretted hydrogen, which will produce a black deposit; lastly, the liquor will have a sugary taste.

*Treatment.* Glauber's salt, Epsom salts, gypsum or well water, are the best antidotes of lead. The patient should therefore be treated as for the swallowing of barytes.

*Colica Pictonum.* It is well known that painters plumbers, and others, whose occupations lie among lead are affected with what has been termed the Painter's Colic, or dry belly-ache. The following mode of treatment has been found very useful in the L'Hôpital de la Charité of Paris: 1st day, A purgative glyster of infus. sennæ with sulph. sodæ, giving the patient for drink a decoction of cassia fistularis with sulph. magnesiæ and tartarized antimony.—In the evening, an anodyne glyster of sweet oil and laudanum. 2nd day, An emetic, six grains of tartarized antimony at two doses, following it with a sudorific potion of decoction of the woods.— In the evening, an anodyne glyster as before. 3rd day, A gently purgative mixture of senna infused in the decoction of the woods. 4th day, A purgative of salts, jalap, and syrup of buckthorn, with senna.—In the evening, the anodyne glyster. 5th day, The gently purgative potion of the second day; the anodyne glyster in the evening. 6th day, Same as the fourth. If these means fail to procure copious fæcal evacuations, the following boluses are given:

℞. Pulv-scammoniæ, gr. x. pulv. gambougiæ, gr. xij. confection of hamech ℥iß. syr. rhamni cathart. q. s. This is made into 12 boluses, one of which is given every two hours. It is very rare that a treatment of this ki d fails of curing the disease.

### Class II. Vegetable Poisons.

1. Irritating Vegetable Poisons, *as Aconite, Colchicum Autumnale, Scammony, Euphorbium, Gamboge, Hellebore, &c. &c.*

These have more or less of an acrid, biting, bitter taste, occasioning burning heat, and great dryness of the tongue, painful constriction of the throat, inclination to vomit and stool, more or less acute pains in the stomach, difficult and accelerated respiration, dilated pupil, great depression, more or less violent convulsions, &c.

*Treatment.* This does not differ, in many cases, from that which is necessary for corrosive sublimate [Sect. iii. 1.] except that there is no advantage in giving the whites of eggs. No emetics should be given of ant. tart. or the like.

II. The Narcotic Poisons, *as Opium, Black and White Henbane, Prussic Acid, Laurel Water, Lactuca Virosa, the Solanums, &c.*

*Effects.* Stupor, numbness, somnolency, a kind of drunkenness, dilated pupil, delirium, convulsions, palsy, full pulse, vomiting, death.

*Treatment.* If the poison have been swallowed, four or five grains of emetic tartar, dissolved in water, should be given; and if vomiting do not take place in a quarter of an hour, 24 grains of sulphas zinci, dissolved in a glass of water, and given in two doses at a quarter of an hour's interval, if the first dose does not produce the effect. If these means do not succeed, three or four grains of the sulphate of copper may be given, dissolved in a glass of water—always with the intention of expelling the poison, either by vomiting or by stool. This should be promoted by introducing the fingers into the throat, or tickling it with a feather. *All acids are hurtful,* instead of beneficial, before the poison be expelled. If the poison have had time to pass into the bowels purgative glysters should be given.

Suppose the patient has vomited entirely, or almost entirely, the poison, the disease might still prove mortal, if left to itself. Here we must alternately, every five minutes, give a cup of water, acidulated with vinegar, lemon juice, or cream of tartar, and a cup of coffee. The numbness must be counteracted by frictions. Blood-letting may be sometimes necessary.

III. Of Poisonous Mushrooms.

These produce griping pains, inclination to vomit, purging, heat of stomach, languor, acute and almost continual pains, cramps, convulsive movements of different parts of the body, intense thirst, small, hard, and quick pulse, intoxication sometimes, with delirium and lethargy.

*Treatment.* No acids to be given until the mushrooms have been ejected by vomiting and purging. Three grains of emetic tartar should be given in a glass of water; and this should shortly be repeated, if unproductive of the desired effect. After copious vomiting, the remains of the poisonous substance should be carried off by purgatives. If these fail, a tobacco glyster should be given, which generally produces vomiting. After the evacuation, cordials should be given.

The subsequent treatment for inflammation, if it supervene, must be conducted on general principles. The above observations will apply to tobacco, belladonna, stramonium, digitalis, hemlock, &c.

### Class III. Animal Poisons.

### *The Viper, Rattlesnake, Scorpion, Tarantula, &c.*

*Effects.* An acute pain felt in the wounded part, soon spreading to the whole limb, and afterwards to the interior of the body. A swelling, at first hard and pale, becomes red and livid, as if gangrenous, increasing and extending by degrees to the neighbouring parts. Faintings, vomitings, and convulsive movements afterwards occur, and are sometimes followed by jaundice; the stomach becomes irritable; the pulse frequent, small, and irregular; the respiration difficult, with cold clammy sweats, derangement of vision and the intellectual faculties. When these symptoms have attained a certain height, inflammation and suppuration ensue in the wounded part, and if the abscess be very considerable the patient dies.

*Treatment* [*external.*] Moderately tight ligature above the bite; wound left to bleed after being washed with warm water. If inflammation be considerable, the ligature will be improper. The wound to be cauterized with a hot iron, lunar caustic, or butter of antimony. A mixture of one part of liq. ammoniæ and two parts of oil applied on the swelled parts around the wound has been found useful.

*Treatment* [*internal.*] Volatile alkali should be taken in any warm diluting drink, to bring out a perspiration if possible. The patient should be placed in bed, and well covered. He may have a small glass of Madeira wine occasionally. Ipecacuan or emetic tartar may be administered. If gangrene advance, the cinchona must be given.

In several countries of America a plant called *Guaco* is employed by the Indians as a remedy for the bite of the numerous serpents which infest those regions. Arsenic internally exhibited has also been found serviceable.

Into the nature of poisonous fishes we shall not enter, since we verily believe that the subject is as yet enveloped in the most impenetrable darkness, into which airy speculation and error only have found their way. In respect to treatment, emetics, we believe, are the only known antidotes, if they may so be called ; after which æther and other antispasmodics, with acids, are serviceable to recruit the nervous system after the shock of the poison.

For the same reason we shall decline entering on the nature and treatment of hydrophobia ; believing the *first* to be unknown, and the second *inefficacious.* As a *preventive* of the rabid poison entering the system at large, we conceive that excision, or complete destruction of the parts, is the chief thing to be relied upon, and next to that mercurial ptyalism, as ascertained by Mr. D. Johnson, many years a medical officer in the East Indies.

We have now presented our readers with a Toxicological EPITOME that may not be unworthy of their *occasional* perusal, and their constant reference in the moment of exigency. We have constructed it so that the eye can catch, in half a minute, a view both of "bane and antidote ;" and if our labour shall prove instrumental in directing with celerity, and consequently with efficacy, the means of relief to even a single fellow creature writhing under the pangs of poison, accidentally taken, or criminally administered, we have our reward.

---

### II.

### *Medico-Chirurgical Transactions, Vol.* ix. *Part* 1.

ART. 1.—*On the Yellow Fever, as it appeared in the Island of Antigua, in* 1816. By A. MUSGRAVE, M. D. of Antigua.

THIS is a very candid and faithful account, both of the fever of the island, and the feelings of the author. For a considerable period previous to 1816 Antigua enjoyed so fortunate an immunity from fever, that people began to wonder where had fled those formidable diseases which in the schools of medicine are held up in terrorem to the young student. In the year above mentioned, however, the unwelcome visitor returned, after a long absence, and evinced its true nature by the same characters in which authors have always clothed it. It attacked the Europeans, as usual, and spared the natives; but of all descriptions of the former, the Scotch felt its direful influence most. Among Europeans it did not make much selection; the full-blooded and the thin, the male and the female, came equally within the sphere of its power. In respect to the point of contagion, Dr. Musgrave joins his voice to the countless host which now stamp so decided a negative on that question. He also adduces evidence of secondary attacks of yellow fever, in opposition to the supporters of Dr. Pym's opinions. He believes that the yellow, or what has been termed the Bulam fever, is only the highest grade of the marsh or remittent fever of the country. The state of the atmosphere presented nothing unusual; the weather was generally fine, with occasional showers; the heat was by no means intense; "and indeed," says our author, "I must candidly confess my utter inability to attempt any explanation recommended by the slightest shadow of probability, why the ordinary causes of fever should at that time have assumed so unusual a degree of virulence." P. 131.

The only thing remarkable was this, that after each day's rain "the crop of cases" was more numerous and severe than before. A sharp gale, amounting almost to a hurricane, in September, produced no salutary influence on the disease.

The symptoms of the fever were those, as described by authors. From the black vomit, Dr. M. never saw but one recovery. A wedge-like sensation at the cardia, or, as many expressed themselves, "as if a marble were sticking there," always excited the utmost apprehension of the approach of this symptom. Visceral obstructions were rare as a sequela of this disease. This circumstance is surely not very favourable to the opinion of its being only a grade of the intermittent and remittent fever, which leaves such constant traces of organic disease in the abdominal viscera. As for treatment, our author observes, that the man who wishes to save his patient must act with decision, or he will fail in his object.—"He must bleed; but he must bleed early, from a large orifice, and till some manifest change is produced in the circulating system." This applies of course to the early stage; but afterwards great judgment, if not great caution, is necessary.

"I would hold it a safe rule in doubtful cases, to be cautious with the lancet after the first twenty-four hours, and throw it aside altogether after forty-eight have elapsed."

"We have repeatedly, with success, taken upwards of forty ounces of blood at one bleeding. With equal success, in several cases we have renewed the bleeding up to the third and even the fourth time." "In our practice, the bowels were always freely evacuated by calomel and jalap, or some other active medicine, in the first instance, and were kept soluble during the whole progress of the fever. But I think I have seen the fatal event accelerated, where, from an over-anxiety in this respect, medicines too harsh were administered at a late period, by which the most debilitating catharsis has been induced, not to be checked by any means that could be devised."

The cold affusions were changed for ablutions, which had a better effect. We were somewhat surprized at the following passage.

As soon as the circulating mass is lessened, and the intestines *fully emptied* [an Hibernicism surely] the administration of bark ought to be commenced. If we longer delay, if we wait for a marked remission, the foundation of the very mischief we wish to avert will have been laid. The powder should be first attempted, but in the event of its oppressing the stomach from its bulk, the combination of serpentaria with it in infusion, adding a proportion of the sp. æth. nit. was generally an admirable form of prescription." P. 138.

To facts we dare not object; but to precepts we may refuse our assent with strict justice. We believe then that our best tropical practitioners will object to the foregoing passage, as veering round to the old system of "throwing in," and as subversive of the sound pathological views which of late have opened upon us in tropical fevers.

Mercury was not used in a salivating character till late in the epidemic, when some army and navy surgeons had recommended it. It was then conjoined with the depletory system, and proved useful.

"In the remittent form, where bleeding has not produced the wished-for effect of giving an immediate check to the progress of the symptoms, I think I have observed the greatest advantage derived from its employment; and I have satisfied myself of the slighter degree of irritation caused both to the stomach and bowels by large, than by more minute doses of calomel. Fifteen grains or a scruple may be given with perfect safety, and the dose repeated every four or six hours, the practitioner, of course, watching the effect, and acting accordingly." 140.

These are the prominent and the practical features of Dr. Musgrave's paper, which is characterized by much modesty of opinion, liberality of sentiment, and no inconsiderable share of judgment.

### Art. 2.—*A Case of Loss of Power over the voluntary Muscles.* By John Bostock, M. D.

Mr. H. ætat. 30 or 40, who had previously enjoyed good health, complained of a pain in one of the lower extremities, on the outside, and a little above the knee, shooting up occasionally to the hip. He had had a slight fall on the back some months before. In two months some loss of muscular power in the limb took place, and the pain advanced higher up the thigh. During the next two months the complaint increased, and his general health became impaired, with loss of appetite, irregularity of bowels, and hæmorrhoidal affection. In two months more, the other limb began to suffer in the same way, and he now felt a painful sensation on first putting his feet to the ground after resting. Shortly after, he perceived a difficulty in articulating particular words, which increased, *pari passu*, with the loss of power in the limbs, till at length he nearly lost both motion and speech. In eight months from the beginning, pain in the back of the head and neck was felt, and soon afterwards stiffness in his hands and arms. In no long period after this, the deglutition became affected, with loss of power over the muscles of the jaws and the neck.

His situation was now deplorable; but he was destined to endure still greater sufferings. He was gradually reduced to a state of complete helplessness. The power of the limbs was almost entirely gone, and the jaws could only be opened just so far as to receive between them a small tea spoon. His head fell down upon his chest, unless when artificially supported; there was constant flow of saliva from the mouth, and he was frequently almost suffocated with mucus, or convulsed with the ineffectual efforts to expel it. To these symptoms were added occasional rigidity of the whole body, pervigilium, &c. Yet the sensibility of the surface and the mental faculties remained unimpaired, and at times he manifested even a degree of cheerfulness.

For some time before his death, the upper extremities, as well as the lower, were entirely motionless, and the only method he had of expressing his wants was by pushing his body against a moveable staff, which communicated with a board, on which the letters of the alphabet were placed, and by varying the degrees of motion, the staff was made to point to the different letters. No relief was experienced from any medicine.

*Sectio Cadaveris.* No morbid appearance could be detected iu the brain, or cervical portion of the spine, except the following, if it deserve the appellation of disease.

"After a very accurate survey of every part, we thought that we observed a slight furrow across the spinal cord, as if it had been compressed by a transverse ligature, and this in the place where it passes under the ring of the atlas; and, upon attentively noticing this part of the bone, it appeared a little thickened, and of a yellowish colour. These appearances, however, were not very distinct, and the change of structure existed in so slight a degree, that it would probably have escaped observation, had any other morbid derangement presented itself to our notice." P. 8.

It may be observed here, in the first place, that though the *voluntary* motions of all kinds were completely interrupted, yet the *involuntary* or automatic motions went on nearly in their natural state. It is quite evident from this, that the only portion of the spinal canal, where the cause of the disease could be reasonably expected, was left unexplored. How could the source of the affection be supposed to exist in the brain, when "his senses, both mental and corporeal remained unimpaired!" P. 6.

Dr. Bostock queries whether the disease might not be entirely an affection of the voluntary muscles, independent of the nervous system—that is, an incapacity, on their part, of receiving the nervous influence, or of being acted upon by it? We think it extremely unlikely, that the one class of muscles (differing in no essential respect from the other; for in what do the intercostal muscles, that act in respiration, differ from the pectoral muscles that lie over them?) should be thus incapacitated, while their companions in the automatic system remained unimpaired; whereas every day shews us, that one portion of the seat of nervous power will be deranged, the other portions retaining their integrity of structure and function. We regret, therefore, that this subject should have been left in doubt by the omission of examining the dorsal and lumbar portions of the vertebral column.

Art. 3.—*Case of Cynanche Laryngea.* By Dr. Arnold.

Mr. B. a farmer, after exposure to cold, complained in the night of a sensation of soreness in the throat, which increased, in a short time, to inability of deglutition, spasms of the throat and chest, and a distressing sense of suffocation. This paroxysm lasted two hours; ten ounces of blood from the arm. No appearance of inflammation or tumour of the tonsils, uvula, velum pendulum palati, or fauces; voice hoarse; expuition of mucus; pain in the region of the thyroid cartilage. Abstraction of twenty ounces of blood; tendency to deliquium; abatement of pain. With great difficulty got down ten grains of calomel, which induced a paroxysm of dyspnœa and sense of suffocation. An enema ordered, and the calomel to be repeated every four hours. Leeches to the throat; blister to the sternum. The purgative glysters and calomel to be continued. On the fourth day, all things were better. The calomel had acted on the bowels; there was a tendency to ptyalism; an equable warmth was diffused over the skin; the appearance of the countenance was good. On the sixth day, the patient was convalescent.

From some experience in this complaint, we beg to say, that the inhalation of warm vapour, and the semicupium, are powerful auxiliaries to the above measures, together with nauseating doses of the tartrite of antimony.

### Art. 4.—*Case of Hereditary Ichthyosis.* By J. P. Martin, Esq.

Jane Holden, aged three years. Her whole skin, except the face, is covered with scales, or rather warty or bristle-like projections, varying in colour from the lightest brown to the deepest black, in some parts having a yellowish tint, as if scorched by the fire. They vary in size and form, in different parts of the body, but are mostly long and flat, and standing at right angles to the skin. They are easily removed when they grow long, and are constantly exfoliating and renewing. The child seems to suffer no uneasiness from this extraordinary state of the cuticle, except a slight occasional itching, and is, in other respects strong and healthy. The disease commmenced, without any previous or concomitant constitutional disorder, when the child was three months old, beginning about the joints, and upon the soles of the feet. It commenced in the mother about the same time. Her cuticle is nearly in the same state as the child's, except upon her neck, bosom, and fore-arms, where it is natural. Her legs are completely encased in thick scales, and, at a distance, might be taken for those of a negro. A drawing, representing, with sufficient accuracy, the appearance of the child, and also a portion of the skin of the mother is given.

### Art. 5.—*On the Transfusion of Blood by the Syringe.* By James Blundell, M. D.

In the Monthly Medico-Chirurgical Journal for April 1817, No. 16, p. 276, we translated Dr. Leacock's experiments on this subject, in which many interesting phenomena were brought to light. Dr. Blundell has improved upon Dr. L's apparatus, by substituting the syringe for the tube, by which means human blood may be transfused into the human veins, and thereby the danger avoided of introducing the blood of one animal into the vascular system of another. Dr. B's experiments are ingeniously contrived, adroitly executed, and clearly described. They are highly deserving the attention of the medical philosopher.

### ART. 6.—*Notes of a Case of Mercurial Erethism.* By T. BATEMAN, M. D.

Mr. JOHN PEARSON appears to have given the first accurate description of this singular affection; yet as its history and nature are by no means well known, even in the upper walks of the profesion, the Case related by Dr. Bateman is well worthy of record, and as such, we shall give its outline here.

The patient seems to have been affected with amaurosis, and some derangement of the chylopoietic organs, for which he was recommended inunction of mercurial ointment, to the amount of a drachm every night. In a few days the gums became affected, when languor and febricula ensued, with a violent and irregular beating of the heart, which did not yield to laudanum and stimulants, but suddenly went off spontaneously. This irregularity of the heart's action, with various febrile and anomalous symptoms, returned several times, and obliged the patient to desist from mercury. The palpitation continued, however, with the addition of a troublesome cough, which was obviously connected with a flatulent state of the stomach, and not of pulmonary origin. This increased very much, coming on in violent paroxysms, and producing retchings, though not vomiting. These paroxysms were generally relieved by spiced wine.

"This languor and debility continued to increase rapidly every day, as well as the cough, which was exceedingly harrassing, and produced a distressing sense of binding, and of immoveable constriction across the region of the diaphragm, which, together with the continuing perturbation of the heart, rendered it necessary to remain in nearly an upright posture even during the night." P. 223.

The nights were now almost entirely sleepless, from the palpitation and cough. Hyoscyamus and the tinct. humuli gave little or no relief. Nutritious food and drink were now ordered, in small quantities, through the day and the night. After very short slumbers, he would start from his couch, demanding fresh air, and being in some mental confusion.

" On the night of Wednesday the 12th, having again obtained a short sleep, not exceeding a quarter of an hour, awoke in extreme distress, with a sensation of sinking, which was felt as that of approaching dissolution; the anxious demand for fresh air was repeated, as well as urgent calls for brandy undiluted, which was greedily swallowed to the extent of three glasses in the course of five minutes, without producing much relief. Ammonia and æther were then substituted, one or other of which was repeated every ten minutes for about two hours, when the faintness rapidly declined, especially after a very copious discharge of limpid urine." P. 226.

All this time the mental powers remained clear, the extremities warm, and the pulse, though extremely irregular, was felt in the different members. It was curious, that the irregularity of action in the heart increased during sleep to such an extent as to be, in fact, almost suspended, and thus to occasion those alarming faintings and sinkings; so that it was necessary, notwithstanding the extreme drowsiness which succeeded the long continued watchfulness, to interrupt the sleep at the expiration of two minutes, by which time, or even sooner, the sinking of the pulse and countenance indicated the approaching languor.

To the above distressing symptoms were next added, inability to take solid food, the most painful distention of the stomach, generally occuring with the spasmodic cough and faintings, with unusual violence soon after midnight, and somewhat abating towards day-break. The greatest relief was found from a musk draught, containing ten grains of that substance. This generally excited a kind of electric tingling, even to the extremities of the limbs, and an immediate feeling of renovated strength. After this, the bark, in decoction and infusion, and subsequently in extract, was given, with light nutritious food, in small quantities, and at short intervals. About this time œdema of the lower extremities became considerable.

" The enfeebled condition of the stomach, and especially the immoveable flatulence which was accompanied by a sensation of globus, being rather increased, as well as general debility, it was this day determined to give up all solid food and medicine, and to limit the diet to asses' milk, to the extent of a quart daily, taken alternately with cows' milk, into which a small quantity of tops and bottoms was occasionally broken. The benefit of this change in the ingesta was most sensible, for immediately the flatulence began to subside, and the harrassing cough to diminish; and from this time a progressive, though very slow improvement of the strength and all the other symptoms took place. The stomach regained its powers by very slow steps." P. 230.

It was remarkable, that during a subsequent fortnight of great vascular irregularity, while the heart beat at 160 in the minute, the arterial pulsations at the wrist were just half the number. Now we believe we can throw a little light on this subject from painful and anxious personal sufferings. Being subject to intermissions of the pulse, we have invariably observed, that at the moment of intermission *in the pulse,* there is a more violent than usual stroke of *the heart* against the ribs. Thus it is quite evident that the heart's action is not intermitted, but that the central organ is unable to cause a propulsion of the blood through the arterial system at those times. The same phenomenon we have observed in many others. Now if it so happen that this *ineffective* effort of the heart occurs at every *second* systole, there will be just double the pulsations at the heart that there is at the wrist, as, in the above case, related by Dr. Bateman. We often hear of want of synchronous action between the heart and arteries:—we have often observed this want of consonance between the pulse and heart; but it was invariably in the manner above—always more pulsations in the chest than in the wrist. In all the varieties of pulse which we have felt, we never could perceive an undulation in the artery, without an action of the heart; but very often an effort of the heart without a pulse at the wrist.

In the end, this gentleman continues slowly regaining his muscular strength, the circulation being hurried, and lassitude and aching of the limbs induced by very moderate exertions in walking, and the pulse at all times exceeding eighty beats in the minute.

### Art. 7.—*On the Effect of Nitrate of Silver on the Complexion.* By Dr. Badeley.

Stephen Martin, a Quaker, eighteen years of age, sensible and temperate, had enjoyed good health till he was attacked with epileptic fits. The first was at the age of fifteen; after which they recurred at uncertain periods, generally every third or fourth week. They appeared to originate in the stomach; he had great acidity there; a favourable symptom in Dr. Badeley's opinion, who states, that a nun in the neighbourhood had epileptic fits almost every night for some years, with the greatest possible degree of acidity in the stomach and mouth. She now has them only twice or thrice in the year. He ascribes the immunity to lunar caustic and magnesia.

Martin's paroxysms left a violent pain in the head, and particularly in the eyes. Between the fits he felt "sensations like flashes, or quick passing vapours behind his eyes, followed by a bewildering feeling, with a violent pulsation in his head, and a temporary deprivation of sight." These sensations were removed with the fits by the use of argentum nitratum, *unassisted*, after leeches, blisters, emetics, mercurials, bark, steel, zinc, valerian, and turpentine had failed. The argentum was taken in doses of a grain to a grain and a half, three times a day, made into pills, with crumb of bread, and continued a year and a half. By this alone, the fits gradually lessened in frequency, till they entirely left him. In other affections of the head, from their apparent propinquity to epilepsy, Dr. B. has exhibited this medicine with success. In all these cases it operated briskly on the bowels, producing four or five motions daily.

"In Martin's case it did not; but I have generally found it most successful when it purged."

"It was remarkable, that in one of these cases, a young man, who was very weak, partly from evacuations, and from having no appetite, although he had four or five motions from his medicine, they did not increase his debility; and he begged of me not to check them, as he felt himself daily getting better. This man had been frequently purged by other cathartics without the least benefit." P. 237.

Dr. B. thinks that this medicine would be more frequently efficacious were it given in small doses, and with sufficient perseverance.

The patient above mentioned experienced the effects now so often ascertained, as resulting from nitrate of silver; viz. a darkened hue of the skin.

"It is now nearly two years, and his face still retains the leaden colour; his bosom rather darker, with a purple hue; the roof of his mouth, inside of his cheek, and back part of his tongue dark; the tunica sclerotica much discoloured."

Blisters rose white, as on Negroes, proving the rete mucosum to be the seat of colour. He has been free from epilepsy nearly two years and a half.

A hint is thrown out by our author, which we think worth recording.

"We know that turpentine quickly communicates its odour to the urine of a person sitting in the same room with it; and many painters have told me, that they do not feel the pernicious power of lead, when they paint with it unmixed with turpentine, but soon feel its deleterious effect when united. A maid-servant of mine lost her speech, and became paralytic from being six hours in a room newly painted, but quickly recovered upon being removed. If, then, turpentine so soon convey the pernicious power of the oxide of lead, may it not in like manner be made the beneficial conductor of the oxide of mercury? May not children, or patients, who cannot, or will not admit mercury in any other mode, receive much benefit in many cases by sleeping in rooms, the air of which is strongly impregnated with turpentine, or with æther, loaded with mercury?"

To the latter suggestion we would object, on account of the uncertainty which must always appertain to the *dose* of a powerful medicine, administered in this way. We know the great difference of absorptive power in different individuals, and this would occasion perpetual hazard in the aerial employment of medicines of this description. The hint relative to turpentine and lead, however, is very valuable.

Art. 8.—*Report of the Principal Diseases in the Royal Military College at Marlow, Bucks, and Sandhurst, Berkshire.* By N. Bruce, Esq. &c. &c. &c.

This Report embraces a period of seven years; viz. from 1809, till 1816. After some pertinent remarks on the Medical Topography of Marlow and Sandhurst, together with the economy of the establishments themselves, Mr. Bruce proceeds to the consideration of the diseases, in the following order.

1. *Fever.* Under this head he includes all those cases in which "acceleration of the pulse, increased temperature of the skin, preceded by the well-known usual symptoms, were the predominant phenomena, without the *manifest* presence of primary local affection."

In the more severe cases, the symptoms were those described as appertaining to *synochus* by systematic writers. In no instance could these be traced to contagion, nor did they ever degenerate into typhus so called. The first therapeutic step was cleansing the primæ viæ well by tartrite of antimony, sometimes combined with ipecacuan, so as to produce vomiting; and as soon as the stomach was sufficiently composed to retain them, calomel with jalap, or extract of colocynth, was given as a purgative, assisting its operation by supertartrite of potass, neutral salts, or infusions of senna, so as to procure five, eight, or ten alvine evacuations, within the first twelve or fifteen hours after the patient's admission.

"To accomplish this object, from six to twenty grains of the submuriate, and from fifteen to sixty of jalap, according to the age and constitution, exhibited in two or more doses, have, in many cases, been found necessary, with the additional assistance of neutral salts and infusions of senna." P. 259.

To accelerate the operation of the cathartics, glysters were often exhibited where the febrile symptoms ran high. An abatement of heat and vascular excitement was thus effected, commonly within twelve hours; if not, the cathartics were repeated.

"It was remarked that repeated active doses of these medicines, so far from inducing debility, too often and too groundlessly dreaded in such cases, have commonly procured to the patient relief from the severity of his sufferings, and an apparent renewal, as it were, of the strength and energies of the system." 260.

When from the inefficacy of these means, their too late application, or any other cause, the febrile action became entirely established, then the medical attendants generally found some lurking local inflammation or affection of the head, or some part of the thoracic or abdominal cavities, as evinced by headache or giddiness; pain on deep inspiration, or on pressure with the hand in the different regions of the body. This discovery led, of course, to the use of blood-letting, general or local, blisters, and other counter-irritants. The extent of the venesection was regulated by the pulse, but especially by the relief of the local symptoms. Even where there were no well marked topical affections, and where the febrile actions had not yielded to the active medicines previously administered, so much as might have been expected, and where the strength and frequency of the pulse seemed to indicate inflammation *somewhere*, "blood-letting has been repeatedly followed by an evident relief of almost all the symptoms."

"In such cases, when carried far enough to produce a very sensible impression upon the general system, this powerful remedy, by its immediate effect of reducing the volume and momentum of the circulating mass, and thus counteracting local congestion, at the same time that it commonly abates the quickness of the pulse and the cutaneous heat, has seemed to open the way towards a more favourable crisis, and bring about a speedier termination of the disease." 262.

When all these means had failed to arrest the progress of the disease, and it had become catenated, as it were, and destined to run a certain course, then Mr. Bruce was in the habit of prescribing "small and frequently repeated doses of the submuriate of mercury, in the quantity of seldom more than two grains, given every four or five hours, in conjunction with the antimonial or James's powder, without opium; and when found necessary, these were joined or alternated with sufficient quantities of cathartic medicines, so as to keep up daily copious evacuations." In the intervals, saline draughts and acidulated drinks were freely used. The fetor and appearance of the stools afforded a very convenient criterion for regulating the exhibition of the purgatives. The temperature of the chamber was also attended to. In respect to the cold affusion, which was tried on the principles of Dr. Currie, our author confesses, that although it repressed for a time the heat and febrile anxiety, and frequently the strength and velocity of the pulse, "yet the consequent reaction of the heart and arteries was very generally attended by a return of the symptoms to the same, or nearly the same degree of urgency, as before the affusion. The patient was, however, generally speaking, much refreshed by it; and although it did not effect any permanent good, it afforded him a respite from feverish heat, and was so grateful to his sensations, that he sometimes earnestly requested it to be repeated." 266.

This corresponds with our own experience, and we fully coincide with Mr. Bruce, when he says, that in this fever of the cadet's, as well as in most other fevers, early and copious evacuations, particularly blood-letting and cathartics, are the most indispensible remedies.

Only two cases proved fatal out of 162, a proof of the mildness of the disease. One of these was a youth, 16 years of age, whose fever was protracted to the twenty-eighth day; during which time, the skin remained obstinately hot and dry, accompanied for some time with a small pearly eruption on the body; the pulse at first 108, afterwards ranging to 140, and upwards. Redness and swelling of the tongue; and in the early stage, tenderness in the epigastrium, with some degree of tension. The cold affusion produced no good effect; venesection was not tried. The body was not examined. The second fatal case was of an insidious nature, and shewed no alarming symptoms till the evening of the 11th day, when delirium very unexpectedly came on, followed by coma and other symptoms of inflammation in the brain, the pulse rising to 140 in a short time. The early and liberal use of purgatives served to keep the disease in check.

Only local blood-letting had been used till the delirium manifested itself. After various means were tried, venesection was now determined on as a dernier resource. About eight ounces of blood were taken away from the jugular vein in a full stream, but the most alarming syncope supervened, and nearly annihilated him on the spot. He rallied a little, and the symptoms of distress were somewhat abated. He died about six hours afterwards.

The brain and meninges exhibited marks of previous inflammation, and six ounces of water were found in the ventricles.

2. *Scarlatina.* In the treatment of this, as well as of fever, the utility of full catharsis, preceded by emetics, was most conspicuous, especially in the beginning. "With this view, the submuriate of mercury was very generally employed, in combination with other aperients, in the energetic manner already described in the treatment of continued fever, and with similarly good effects. The rest of the treatment was generally conducted in the same order and manner." 274. Here the external use of cold water was found very beneficial. Counter-irritants were applied to the throat, with large hot cataplasms to the external fauces.

3. *Epilepsy.* Seven cases of this disease occurred, most of which were of a slight nature, requiring little other treatment than evacuations. The cold affusion was useful in one case.

We have now concluded a very copious analysis of the Medical articles in this volume. The Surgical and Parturiological papers will be noticed in their respective departments of the Journal.

---

### III.

*Observations on a Stridulous Affection of the Bowels; and on some Varieties of Spinal Disease: with an Appendix of Cases.* By J. Bradley, M. D. Octavo, pp. 283. London, 1818.

We have reason to believe that the circulation of a book, and particularly a medical book, is considerably influenced by its title. That of the work before us, though perhaps a very proper, is a very unpromising one, and we *know* that this very circumstance has checked its sale. We shall therefore endeavour to give such an analysis of the volume as will enable the reader to appreciate its value, without any other criterion than his own judgment.

The peculiar noise in the bowels implied by stridor abdominalis, though not confined to either sex, is most peculiar to young females, from the age of twelve to thirty years. Anorexia and dyspepsia either precede or accompany it, from its first attack, together with chlorotic paleness—some degree of emaciation—lassitude. To these symptoms are occasionally added head-ache—pains in the loins and lower extremities. The pulse at the onset, or during the first stage of the complaint is natural, but becomes accelerated towards evening afterwards. Slight rigors, or almost a constant sense of chilliness is prevalent in the day-time, succeeded towards night by some heat, without much increase of the secretions. Some symptoms of gastric derangement accompany the above, with high-coloured urine and sediment. The bowels are constipated, but the fæces not morbid.

"The noise within the abdomen returns at uncertain intervals in the course of the day, and is of no limited duration. The period of its continuance, however, seldom exceeds twenty minutes, or half an hour; and always in this stage, while the patient is in an erect posture; for on lying down, it will almost instantly cease, and be no more heard, as long as the body is in an horizontal posture, during the usual time for rest. Whatever is taken into the stomach, whilst the body is erect, has no inconsiderable influence in exciting or abating this stridulous sound. For instance, after the patient has sat down to a meal, and taken a few mouthfuls, it will invariably ensue, and continue for some time; after which, it becomes weaker, and more and more intermits till it ceases. On the contrary, however, instead of food producing this effect, it often abates this noise, especially when the stomach is empty, and there is faintness, with a sense of craving for food; which is sometimes most prevalent on rising from bed in the morning, about eleven o'clock in the forenoon, and at five or six in the evening. This noise, which is always under the governance of respiration, is, for the most part, similar to the croaking of a frog, especially on inspiration; but on expiration, somewhat less so, and conveying an idea, as if the sound issued from water, and often, before it ceases, like the plaintive tone of a dying animal." P. 6.

If a hand be placed on the left umbilical region, during inspiration, or near the navel, a sensation is felt as if some liquid was forcibly spirted, or dashed against the peritonæum. If deep pressure be made here, great pain is felt in the region of the stomach, sometimes even to cause fainting. There is seldom either tumefaction or discolouration to be observed in these parts of the abdomen; but the hypogastrium is generally swollen, and pain is felt, when the fingers are strongly pressed on each side of the spinous process of the third or fourth lumbar vertebra from above.

The above described symptoms, though insidiously undermining the constitution, often continue a considerable time, with little apparent increase, and without any alteration in the structure of the spine: but the patient will at length experience a systematic derangement and debility. His complexion will become more sallow, or exanguious, and his spirits more depressed. The spine, at the place above-described, will now be found giving way, and either slightly projecting anteriorly, or to the left side, taking further lateral or oblique directions till it comes to press upon the posterior part of the ilium, and cause the anterior spinous process of that bone to protuberate a little in the same direction. This occasions the right hip to appear enlarged, by reason of the hollowness between the ilium and vertebral column being increased. This lateral distortion also, by dragging with it that portion of the abdominal contents which is more immediately attached to the spine, causes the left hypogastrium to appear more swollen than the right, especially in the evening, when the distortion is generally greatest, from the erect posture of the body during the day. Although the patient may still be able to lie easiest upon this than upon the right side, yet the ribs will be found protuberant; and soon after this, from increasing distortion, the patient will be incapable of lying on either side, from pain in the part. Sometimes the spine takes a counter turn, and pointing upwards, raises the right scapula, together with the superior and subjacent ribs; and, if the distortion be considerable, the head and cervical vertebræ will incline somewhat to the left or adverse side. Sometimes the distortion will advance directly forwards, and remain stationary for a long time; in which case the muscular substance of the sacro-lumbalis and longissimus dorsi muscles, on each side of the lumbar vertebræ will be elevated above the spinous processes, so as to form a deep and narrow sulcus between them.

If the distortion continue to proceed laterally, the symptoms of organic derangement become more pressing. The patient now begins to throw up his food, often attended with pain and uneasiness, or tightness across the stomach. Some pain is also felt in micturition.

Feverish accessions now take place, with various symptoms of derangement and irritation in the system; after which dyspnœa and cough, with hæmoptysis or hæmatemesis, in general, supervene, succeeded or attended by diarrhœa, alternating with partial night sweats, particularly about the head and breast. The urine is now high-coloured, and loaded with a muco-purulent sediment. It is needless to say that from this, till the final separation of mind and matter, the unhappy sufferer presents a spectacle of emaciation and debility which is shocking to behold.

"The noise within the abdomen, which generally continues to increase, will often, in proportion to the distortion, be farther augmented, both in sound and duration; and, as soon as the patient, from weakness, is incapacitated from remaining erect, it will, in this advanced stage, frequently return, even while he remains in the horizontal position." P. 14.

*Causes.* The remote cause of this disease may be traced, in general, to a scrofulous disposition, combined with a tendency to spinal disease, from the natural conformation of the body. The proximate cause is less clear, but generally depends, according to our author's opinion, "on a certain part of the spine in a diseased state, occasioned from constitutional, but more particularly from mechanical agency. And, as a proof of the close connexion of cause and effect between the spine and stridulous noise within the abdomen, I have never seen the latter to exist, when tolerably well marked, for the last seven years, when the former was absent, or without its having previously existed, excepting in one case; but the former sometimes exists when the latter is not present." P. 28.

"Females who are under boarding-school discipline, about the age of puberty, and rigidly subjected to it, by being compelled to sit many hours in the day with the head erect, and the shoulders thrown backward, are obnoxious to it; especially if the circumstance of having a long spine and a large head, chest, and upper extremities, be present. Hence the sufferer can scarcely fail having pain in the loins without the means of relieving it; and if we take into account the abominable custom of abridging these unfortunate victims to boarding-school avarice, of the quantity of nutriment which nature more particularly requires at this period of life, we may cease to wonder either at the disease, or distortion in the loins, which accompanies this affection, being the consequence. Not only this long-continued posture, to which the discipline of a boarding-school subjects young females, but any occupation where the lower part of the spine is bent forwards, and the shoulders at the same time thrown backwards, with an increase of the superincumbent weight, will dispose to this complaint. Hence we sometimes find young girls, who are nurse-maids, and of a slender form, that are in the daily habit of carrying heavy children in their arms, or those who have borne weights on their heads, are subject to this malady." P. 29.

Our author has not seen more than four well marked cases of this complaint in the male sex. Three of these were about the age of puberty, and of delicate constitutions when first attacked. In one a very large tumour, apparently of the encysted kind, formed in the left side, exactly at the point where the verberation was most perceptible; and which, after seven months, broke, and discharged about sixteen ounces of purulent matter. Soon after this colliquative diarrhœa and night-sweats hurried the patient to his grave. In the second case, there were evident signs of internal suppuration, with a similar train of hectical symptoms.

The borborygmus of hysterical females may be easily distinguished from the stridor abdominalis:—the former having a guggling sound, which is perhaps the most frequent when the patient is in bed; whereas the latter is always governed by the action of the diaphragm, and seldom perceptible when in a horizontal posture, and therefore easily discriminated from the former. We often, however, meet with a slight croaking of the bowels for a few minutes, which returns frequently, among women troubled with flatulence, and who wear tight stays; but this may not be connected with any spinal disease nor systematic derangement; and may be considered as spasmodic, though under the control of respiration. It is, our author believes, sometimes a prelude to stridor abdominalis, and often an attendant on other spinal affections, especially on distortion, or displacement of the upper lumbar or lower dorsal vertebræ. The beating in the abdomen can scarcely be mistaken for the abdominal pulsation mentioned by Haller and others.

*Thoughts on Spinal Affections in general.* Under this head, Dr. Bradley makes several pertinent observations on the *mechanical* causes of distortion of the spine. He regrets that Mr. Pott, who had such ample opportunities, "had no idea of the frequent displacement of the lumbar vertebræ." He draws the following corollary, deducible from observation, namely, "*in proportion as any part of the spine diverges, or recedes from its centre of gravity, or is*

*the more mechanically acted upon, it is the more liable to distortion.*" If this be admitted, he thinks that mechanical agency has frequently no inconsiderable share in the production of this malady.

"And besides, the unexpected amendment in the patient's general health, as well as in external appearance, too sudden to be ascribed to the absorption of carious bony matter, on the superincumbent pressure being removed, is another evidence that indisputably proves the frequent existence of this cause, and the necessity of guarding against it." P. 35.

*On mechanical Agency in the Cure of Distortion.* Our author supposes that the frequent endeavours of the patient to adopt such postures as are likely to afford himself relief, most probably suggested the principle of such mechanical contrivances as have hitherto been resorted to. These contrivances have been various, but the most effectual, natural, and easy method, is the horizontal posture, which takes off the superincumbent weight from the diseased part.

"It is really surprising, in some cases of distortion, when we havereason to ascribe the efficient cause to mechanical agency, to observe the happy effects of lying in bed; especially if attended with symptoms of febrile excitement, accompanied with irritation, and derangement in the thoracic or abdominal viscera; as insensible perspiration is thereby increased, the body becomes of a more equable temperature, and the rest of the suspended secretions are now restored. The appetite also returns, and the patient grows plump and taller, with a consequent diminution of the curvature." P. 39.

Dr. Bradley conceives, that a disease of the spine may exist under two opposite states, which he denominates active and passive. The symptoms attendant on the *active,* indicate general or local irritation, as slight accessions and remissions of fever; pain on pressing the curvature; pulse accelerated; urine high-coloured, with mucous or lateritious sediment. The distortion here is not large—forms an acute angle, with considerable compression of the medulla spinalis and its consequences. But the most pathognomonic symptom of this variety is a furred tongue, especially towards the root, and generally exhibiting a yellowish tinge, with little or no thirst. In the *second* variety there is more general laxity of fibre, with a soft, and often a less contracted pulse; the urine is paler, and mostly without sediment; the tongue also is cleaner, and there is no thirst. The curvature is larger, more obtuse, and without pain. The paralysis in the lower extremities is less considerable.

In the first, or active variety, as the grand object will be to abate chronic inflammation and fever, caustics will display their best effects, especially if the progress of distortion be arrested, and no serious inroads be made into the constitution.

"Notwithstanding the good effects of caustics in this variety of distortion, yet the frequent application of a blister will be attended with equal, if not with superior advantages; as it produces, *a priori*, more clearly a double effect, not only by affording a discharge from the part, but, by every new excitement of pain, giving a fresh stimulus to the nervous system." P. 47.

Leeches or cupping should precede both caustic and issues, if there be pain or soreness in the diseased part on pressure. But it must always be remembered, that the removal of pressure from the diseased portion of spine is the *sine qua non*, whether that removal be effected by mechanically sustaining the superior parts, or by horizontal position.

*Spinal Disease, unaccompanied by Change of Structure.*—The lower part of the dorsal, and the whole of the lumbar vertebræ, as well as the sacrum, are much exposed to sprains and external injuries. The immediate effects of these vanish, and a long interval of undisturbed health, with the exception of some transitory pains in the part, on catching cold, may ensue. But ultimately, the patient is freqently seized with a new order of symptoms, which are often considered as having no relation to the real cause. Hence a secondary disease supervenes, which assumes such an anomalous character as no experience or physiological acumen is always competent to unfold.

At the commencement of this new affection, which is generally called forth by a scrofulous taint in the system, the patient complains of dyspepsia, with languor and mental despondency. The countenance assumes that sallow, exsanguine appearance so common in spinal disease. Slight fever accompanies these symptoms; the tongue is foul, especially near its root, and the patient is chilly and susceptible to cold. Sometimes, however, the complaint is ushered in by a smart attack of fever, supervening after some lapse of time, to injuries done to the spine. The pain consequent on these spinal injuries is often so trifling as scarcely to be felt, except when the patient has fatigued himself with loco-motive exercise, or been some time in an erect posture, especially if at the same time the super-incumbent weight be increased, either by carrying burthens, or by manual exertion. The proper remedies here are topical bleeding and caustics, to the use of which a certain reaction of the vascular system will ensue, indicating a diminution of the latent disease.

"Sometimes, when the seat of the disorder is in or about the lowest dorsal vertebra, pain or soreness is felt in the right hypochondriac, epigastric, and umbilical regions. The abdomen is tumefied in consequence of wind and costiveness, attended with a slight degree of soreness and discolouration; which, together with a defect of nervous energy, constitute a train of symptoms that is mistaken for hypochondriasis. Under these circumstances there will be sometimes a slight ptyalism, which is no very uncommon symptom of spinal disease, particularly of the dorsal and lumbar vertebræ. There will also be occasionally about the clavicle, a dull pain, and the tunica albuginea will exhibit a slight icteric tinge. The urine will be somewhat high, and inclining to the orange colour, but seldom containing any bile. The bowels here are also constipated, but the stools appear natural, though occasionally dark or ash-coloured. From a collective review of these symptoms, the practitioner may be apt to suspect some disease of the hepatic organs, and advise the use of mercury; but, on trial, he will find no advantage from it, either in small doses as an alterant, or in saturating the system with it. P. 81.

When this disease occupies the uppermost lumbar vertebræ, it will sometimes simulate nephritis, or calculus, or lumbago, but may, in general, be distinguished from these affections by attending minutely to the symptoms which are peculiar to them. When the species of chronic or sub-acute inflammation is confined to the lowest dorsal, or the uppermost lumbar vertebræ, and not attended to in its incipient stage, the consequence will sometimes be either lumbar or psoas abscess.

There are few affections, as our author justly remarks, to which the human body is subject, that are more obscure, or more frequently mistaken, than chronic inflammation of the spine, unattended by any displacement of the bones. 1st. Because little pain is usually felt at the place of mischief. 2d. Because the irritation is often in the parietes of the abdomen, in a transverse direction with the disease in the spine. 3d. Because there is frequently a sympathetic pain on one side of the head, which is often so very distressing as to engross the attentention of both patient and practitioner. 4th. Because dyspepsia, pyrosis, vomiting, tightness across the præcordia, and other sympathetic affections contribute to mark the real seat of the disease. Dr. B. thinks he has observed the pain in the head, in general, confined to the right side, especially when the spinal disease was in the left. It often begins just below the mastoid process, and passes in the direction of that part of the temporal bone hehind the ear, extending itself upwards, so as to occupy the right side of the occiput. In the night there is commonly a remission of the pain, but it returns with increased violence in the morning. It ultimately affects the power of vision. A part that is very liable to these sympathetic pains is a situation about, or at a short distance from the anterior spinous process of the ilium, and mostly on the left side. The epigastrium, however, is the most frequent seat of the sympathetic irritation, when the primary disease is in the opposite part of the spine. The whole of these sympathies are not only painful, but so embarrassing as entirely to engross the attention of patient and physician. Hence the sufferer has, as we have often known, been harrassed by a long-continued course of severe treatment, without gaining any permanent advantage; whereas, had remedial measures been directed to the immediate source of the mischief, a cure would, most probably, have been obtained.

The sacrum is often the seat of the disease, and requires no ordinary discrimination to detect it, particularly in its nascent state. The prominent features of the symptomatology in this case are, according to Dr. Bradley, pain in the sacrum; pulse small and contracted, not much accelerated; anorexia; languor; constipation of the bowels; tongue more or less coated; sense of chilliness or rigors in the day time; slight degree of pain and weakness in the lower extremities; sometimes tenesmus, or uneasiness about the rectum. This state will continue for a longer or shorter period, when there will be a great aggravation of all the symptoms. This aggravation is often the precursor of suppuration, forming either externally or internally, attended with caries of the bones. If the matter form outwardly, in young subjects, recovery may take place; but if internally, and after puberty, death is generally the result.

"In this country, where scrofula is so insidiously prevalent, we often detect diseases of the spine, such as form the subject of this Chapter, by attending to their sympathies, which have been treated as idiopathic affections, to the distress and negative advantage of the sufferer. Dyspepsia, which is symptomatic, and often accompanied with those sympathies of the head already described, and which is often thought to be the occasion of them, is considered as idiopathic; and, when the tonic plan of treatment is adopted in consequence, it is mostly productive of injury, by increasing systematic as well as stomachic derangement. When, however, this effect is produced by tonics, there will always be ground, under these circumstances, for suspecting some spinal affection." P. 93.

*The Disease in Children.* When this affection is attacking the spine of a child, the mother or nurse will observe that the little one is more dull than usual, is weaker, and more disposed to be quiet than on its legs. She will also observe, that the child seems uneasy in certain positions, particularly when laid on the lap. To detect the seat of the disease requires some address. The surgeon should run his fingers gently along both sides of the spinous processes of the vertebræ; and he must do this several times, in order to give the child the idea of being played with. After gaining the confidence of the little patient, he may then venture to increase the pressure, which will soon point out the diseased part, evinced by the shrinking or crying of the child. The examination is again to be repeated along the whole spine, with the addition of some little pressure uniformly applied; and if the child complains most at the same point, which is often between the middle dorsal and middle lumbar vertebra, the practitioner may then conclude that he has discovered the seat of the disease. Some fever and a furred tongue occasionally accompany this affection in children. Here Dr. Bradley glances at a certain species of paraplegia, or paralysis of one or both of the lower extremities, which is mostly peculiar to children about two or three years of age, and neither attended with distortion, nor proceeding from any known cause; as the little sufferer is generally in apparent good health previous to the attack; but which is succeeded by symptoms of reaction, such as thirst and a furred tongue, with increased heat and perspiration in the night.

"I have also observed, that in proportion as the lower extremities are paralysed, the patient will exhibit signs of animation and bright intellects; and hence he always excites peculiar interest and commiseration, on beholding him dragging behind him his dangling limbs, and perhaps, heading a little troop of playful companions, and directing all their childish evolutions." P. 96.

This is a very curious phenomenon, but seems to be accountable on the well known principle that when one organ is functionless, another organ will become capable of increased action.

In proportion as the limbs become shrivelled and flabby, all the rest of the body will be directly increased in muscular energy.

*Cure of Disease of the Spine without Distortion.*—At the onset of the disease, and before much progress is made, repeated abstractions of blood from each side of the spine, by cupping in adults, and leeching in young children, will often supersede the necessity of any farther local means; but when the complaint has advanced farther, a more permanent drain is necessary to prevent a return of the complaint.

"Caustic issues or a copious drain are therefore our principal dependence, and, if timely had recourse to, will be productive of the happiest effects; but to ensure which, it will be necessary, not only to make the caustics penetrate deeply, so as entirely to destroy the cutis, but so extensive as to comprehend, if not the whole, a very considerable portion of the integuments covering the subjacent disease; the propriety of which will be seen on the well known principle, namely, the effects of a drain on local disease being always inversely as to its distance." P. 101.

Our author is decidedly in favour of large caustics, and he objects strongly to the premature removal of the slough, as it deteriorates the discharge, and the sore is thereby rendered foul and unhealthy, from part of the eschar remaining, and which will be slower in digesting off, than if the slough had never been interfered with. As soon as a copious and healthy discharge is obtained, all symptoms of local and general irritation subside; and in proportion as this is kept up, the patient will, in general, progressively recover; but if this discharge, from any cause, be suddenly diminished before the disease be subdued, and which is sometimes the case, the effects are soon perceived by the failure of appetite, and a partial recurrence of the general symptoms. Although the caustic eschar ought, in general, to be allowed to slough off without the intervention of art, yet as some application must be employed, the common resin ointment, either alone or mixed with spermaceti ointment, spread thinly on lint and covered with adhesive plaster, may be used.

"I have seldom made caustic issues in adults, in any species of spinal affections, less than two inches and a quarter in length, and one inch and one-third in breadth, and sometimes of greater dimensions; and when the disease is confined to one, or even two vertebræ, these prove excellent drains." P. 106.

In addition to the use of these drains, the medicines already recommended in cases of local and general excitement will be proper; and after the inflammatory symptoms are subsided, some mild tonic may be given. Bark, however, or any other stimulating tonic, should be ventured on with caution, for as long as the tongue exhibits fur, we ought to be sceptical as to their propriety. When, however, the appetite ceases to improve under the use of these drains, and the tongue is but little furred, the pulse remaining soft and languid, and the urine little changed from the healthy state, tonics may be ventured on.

"Although copious drains are strongly recommended, yet they are not always successful. When the pulse is under a hundred, the greatest advantage may be expected from them. When it ranges from this number to one hundred and ten, their effects are slow, and more uncertain; but if still farther increased, and the tongue display a thick and brownish fur—and especially if there be any visceral disease, attended with colliquative discharges, copious drains are seldom advisable, as they are productive of no permanent advantage, and sometimes are injurious, and consequently may expose the young practitioner to much censure." P. 108.

As another mean of cure, it will always be advisable to take off the superincumbent pressure, on the principle of abating irritation, by either adopting altogether the horizontal posture, or some well-adapted mechanism. In the first stages of the disease, patients will seldom submit to these restrictive measures, but in the advanced stadia the remedy is indispensible; for if caries and consequent distortion ensue, after the age of puberty, the patient generally dies tabid.

We have now given a very full analysis of the didactic and descriptive part of Dr. Bradley's Work, two-thirds of which is composed of an Appendix of Cases illustrative of the foregoing precepts. Of these cases, which are very numerous, we shall not attempt to give any account, but refer to the work itself.

Dr. Bradley is evidently a man of accurate observation, and patient indefatigable investigation. Perhaps, however, he has been too prolix, both in his descriptions and cases, which indeed is the common fashion, or rather failing of the times, and is therefore excusable. The work is a very valuable addition to spinal pathology.

### IV.

### FEVER IN IRELAND.

I. *Report of the Hardwicke Fever Hospital, for the Year ending the* 31*st of March* 1818, *including a Brief Account of an Endemic Fever in Dublin.* By John Cheyne, M. D. F. R. S. E. &c. *Dublin Hospital Reports, Vol. ii. p.* 1 *to* 145.

II. *Medical Report of the Fever Hospital and House of Recovery, Cork Street, for the Year* 1816, *with some Account of the succeeding Epidemic.* By William Stoker, M. D. &c.

*Transactions of the Association of the Fellows and Licentiates of the King's and Queen's Colleges of Physicians in Ireland. Vol ii. pp.* 397 *to* 472.

III. *Medical Report of the Fever Hospital, Cork Street, Dublin, &c.* By F. Barker, M. D. &c. &c.

*Transactions of the King's and Queen's Colleges, Ireland. Vol. ii. p.* 512 *to* 560.

IV. *Medical Report of the Sick Poor Institution, for the Year* 1817. By John O'Brien, M. D. &c. &c.

*Transactions, as above, p.* 472 *to* 511.

Before entering on the immediate subject of the present Article, we beg to congratulate the profession at large on the display of talent, observation, and enquiry, which the metropolis of the Sister Island has lately disclosed, through the medium of the two periodical works now under Review. These works are not only equal to any of the kind which Great Britain ever produced, but we feel little hazard in asserting, that they are *unequalled* by any similar publications which, in the present era of enlightened research, are periodically evolved from the press. We hail this salutary rivalry in medical science as a propitious omen; and were it not that "comparisons are odious," we would invoke the intellectual city on the hills, to shake off her inglorious slumbers, and contribute her mite to the general good. Is the northern star of medicine diminishing in lustre, like its own Auroræ boreales? Has the vivid excitement of BRUNONIANISM terminated in irremediable *exhaustion*—or rather collapse? Are the ORGANS of medical science, beyond the Tweed, *congested;* and has the HEART itself lost its pristine power of maintaining a vigorous *circulation* in the remotest branches of the system? For a school of medicine to support a name, it is necessary that it shall have *writers* who can speak to the profession abroad, as well as *teachers* to address its students at home. We throw out these hints as gentle stimuli to a torpid limb; not doubting that the accumulated excitability will kindly receive the electric spark, and quickly kindle into vital action.

But to return to our proper duties. FEVER, at this moment, is unquestionably the paramount object of solicitude. We had fondly hoped, that in the suppression of war and all its calamitous concomitants, Death had been deprived of one of his arms; but we find his relentless scythe in full play—not mowing down and "gathering unto their forefathers," the aged and infirm, but prematurely shaking the tree of life, and hurling from its branches the tender and unripe fruit. But, perhaps, even the present epidemic might be traced, directly or indirectly, to some of those remote but natural consequences which eventually flow from a long and exhausting conflict with neighbouring nations, aided by one of those inscrutable constitutions of earth and air, which modify and mould the fevers, and other diseases of the period, into their own shape and tendency. In short, the opinion of Sydenham on this subject begins once more to prevail, and it seems destined, that no sooner have we investigated the nature and treatment of an epidemic fever, than all our labour is lost by the capricious changes which it undergoes. Thus descriptions of fever are like descriptions of clouds—a breeze of wind will reverse the whole aerial picture, and so will a few revolutions of the moon round our planet, render nugatory the ablest treatises on fever. Our labours then, on this point, are like the labours of Sisyphus—we have no sooner rolled the stone to the top of the hill, than down it comes again, and the task is to be renewed.

We are among those, however, who think that, "every thing which happens is for the best." The endless labour calls forth unceasing exertions; and thus have talents and industry a never failing scope for theirpowers, and a sure reward for their toils.

Our object then is to accurately analyze each epidemic as soon as possible; to discover in what particular features it differs from its precursors, and what are the modes of treatment which best suit its nature. With this view we shall, in the present Number of the Journal, present the reader with a sketch of the various reports drawn up by our distinguished brethren in Ireland, leaving the fever, as reported on by the English writers, till our next publication.

Dr. Cheyne's Report of the Hardwick Fever Hospital, for the year ending on the 31st of March 1818, is drawn up with the usual talent, candour, and perspicuity of that distinguished physician, and with it we shall commence.

In the above period, Dr. Cheyne himself made daily minutes of nearly 300 cases of fever, and superintended many dissections. His opportunities for observation have therefore been great, and his opinions are consequently entitled to profound attention.

In the year 1817 as in 1816, the order of the seasons, in Ireland, was inverted; the winters in both being remarkably mild and open, the springs ungenial, and the summers wet, cloudy, and cold. The employment, provisions, and fuel of the poor were scarce.

In many cases the fever seemed to spring from contagion. Some patients felt an unaccountable dejection of spirits for several days before seizure; but, generally speaking, there was nothing unusual in the commencement of the disease. There were rigors, confined bowels; inappetency, head-ache, sometimes vertigo, dryness of the nostrils, severe pains in the loins and back, with debility. These symptoms were soon followed by increase of temperature, a degree of fulness of the features, and flushings of the face. The tongue, at the beginning, was generally white, and slightly swollen, with florid edges.

During the spring months, pulmonic irritation, or inflammation was the predominant symptom. In the severe cases of this fever, about the end of the first week, or beginning of the second week, rarely sooner, the patient's minds became unsteady; their eyes suffused, with faultering voice and wandering. This state of restless delirium, in the severer cases, sooner or later degenerated into sopor, and inability to protrude the tongue. After continuing in a soporose state, with partial intermissions, for a longer or shorter time, generally two or three days, the bowels being soluble all the while, sleep became calm and natural, with considerable intervals of waking during the day. As the soporose state went off, the blackness and dryness of the tongue went off also; the expression of the patient daily improved; the temperature fell; the flushing, and the inflammation of the eyes subsided; the complexion became clearer as well as paler, and the eye more expressive. The patient, soon after this, turned upon his side, and about the end of the second week, in many cases, began to appear sensible. When the fever was severe, however, these favourable changes did not take place till the end of the third week.

When delirium set in, the symptoms of pulmonic irritation and also the head-ache, often subsided; and when reason was restored, in a few cases, the pulmonic affection recurred. Amendment was oft times gradual, "without any crisis *but by stool,* unless sleep could be counted critical." In twelve or fourteen days after the crisis, the patients were fit to be discharged.

There never were so many petechial fevers in Dublin, as in the summer of 1817—and this without any connection with the heat of the patient's body, or any particular regimen or mode of treatment.

In respect to prognosis from the state of the vital functions, the breathing afforded the best criterion; next to that the pulse; and last of all, the temperature of the body. Among such patients as were admitted early, and treated on the strictly antiphlogistic plan, there were many instances of crisis on the third or fourth day, especially where the patients came from houses which were not much infected; but the disease was fatal in a large proportion, among such as were received from foci of contagion, and relapses were frequent. Still, "the fevers which we supposed arose from contagion, and those which seemed to originate in intemperance, cold, fatigue, &c. in which we could discover no trace of contagion, were so shaded into each other, that it was impossible, by their symptoms, to demonstrate any difference between them." 16.

The most perfect crisis, during the summer months, consisted of three stages. *First,* a state of restlessness and anxiety, with flushing of the face, rapid pulse, frequent, laborious breathing, and increased heat of surface, with great distress at the pit of the stomach from heat, tenderness, or pain, relieved occasionally by vomiting. This state the nurses well knew as prelusive of the

"*cool,*" or crisis. *Secondly,* a rigor or tremor, not unlike the cold fit of an ague, the patient shivering and complaining of excessive cold. *Thirdly,* warm perspiration flowing from the whole surface of the body. This, in general, completed the salutary effort.

In respect to the morbid appearances, on dissection, the abdominal viscera were found, in general, sound during the first five or six months of the year reported on;—indeed, there were no symptoms, until the middle of August, that indicated acute disease in that part of the system. From symptoms of pulmonic irritation, however, it was expected that the lungs would have been found inflamed; but this was not the case. The expectations of Dr. Cheyne were never disappointed as to the state of the brain, excepting that the diseased appearances in that organ were not always proportionate to the severity of the symptoms of cerebral disturbance. The vessels of the head were found turgid; there was increased vascularity in the brain, especially on its surface. In a few there was slight extravasation of blood from the vessels; in others, serous effusion on the surface, in the ventricles, or into the theca vertebralis, not to great extent.

Dr. Cheyne, though contrary to the opinion of many respectable physicians, believed this fever not to be typhus, but the common continued fever which generally prevails, more or less, during the summer, in many of the great towns in these countries. The indications, he thinks, were obvious, and the remedies such as are in general use.

During the first ten or twelve days, the treatment was strictly antiphlogistic. In those which terminated before the end of the second week, it was generally antiphlogistic throughout. First, the bowels were well purged, and then, in all the milder cases, the disease was left to cold water or whey; cool air, and sponging the head, neck, and chest, with vinegar and water; together with a purgative, when there was not more than one stool in the day. In the more protracted cases, the cordial plan of treatment gradually succeeded the antiphlogistic; provided there was no inflammatory determination, from four to eight ounces of Port wine were allowed daily. On the eleventh or twelfth day, provided the cough was subdued, or had become moist, and there was no head-ache or flushing of the face, or tenderness in epigastrio, our author ordered wine, on the patient's complaining of weakness, or appearing from his position or other symptoms, to be labouring under debility. Along with wine the calomel bolus was given, generally every second day.

In the advanced period of the fever, when there was no local pain or fullness of the hypochondria, especially when the tongue was moist, though not clean, opium combined with mild purgatives, appeared to be more useful than wine.

In April, May, June, July, and August, one hundred and forty-nine were bled out of three hundred; some of them three or four times. Out of these, immediate relief after blood-letting was experienced by ninety-four; "but I am convinced (says Dr. Cheyne) that a much greater number were in an improved state on the day after they were bled." Yet the blood drawn was not sizy in one case in twenty, excepting the relapses, and those cases in which blood was drawn to relieve the inflammatory affections occurring chiefly during convalescence.

The indications for blood-letting were, an increase of vascular action in any of the viscera, such as severe headache, in which case the temporal artery was opened; pain in the chest; dry cough and expectoration of bloody mucus; epigastric tension and tenderness. "Bleeding did not appear to be injurious in any one instance in which it was performed in my wards." As the season advanced, the pneumonic symptoms bore a less proportion to the cephalitic. Although a preference was due to arteriotomy, yet, leeching the temples in the early part of the fever, often succeeded completely in relieving the headache; nay, in some cases, it appeared to carry off the fever in the course of the ensuing twenty-four hours.

Our limits prevent us from going farther into the tables, cases, &c. in this highly important document; or entering on the very valuable Report of the Fever Cases admitted into the King's Military Infirmary. They are all worthy of the able physician by whom they are penned, and do infinite credit to his talents and discrimination.

We now come to Dr. Stoker's Medical Report for the year 1816, in the second volume of the Transactions of the Association, &c.; a great deal of which being taken up with local economy, we must necessarily pass over, concentrating our attention on the purely medical part of the document.

Dr. Stoker conceives the following to be a natural division of continued fevers, as well as a simple and practical one. 1st. Idiopathic, comprehending those of spontaneous and contagious origin. 2d. Inflammatory fevers; and 3dly, a combination of the two first. The characteristics of the first class very generally are, sudden prostration of strength; failure of mental and bodily power; suspension or disorder of the secretions; irregular vascular and pulmonary action, producing both inequality in the distribution of the blood, and of the temperature in different parts of the system, " and a uniform tendency to perform a certain succession of salutary or morbid changes in definite periods." The distinguishing features and local origin of the second class, or inflammatory fevers, are well marked and generally known; but when their symptoms mingle in epidemics with those of typhoid or idiopathic fevers, the importance commensurates with the difficulty of ascertaining their relative degrees, so as safely to decide on the means of cure.

" The opinions on which this division rests, that typhus and synochus are not essentially inflammatory; but that, in their simpler forms, they are diseases of debility, through their whole course, and that the excitement so observable in their early stages, is *constitutional* reaction, accord with my experience." P. 429.*

Dr. Stoker enters his protest against the doctrine of typhus, or what is called idiopathic fever, being dependent on local inflammation, as of the brain for instance, according to the late and present cerebral doctrines. In support of his opinions, he brings forward a very valuable report from Mr. J. Kirby, the director of an anatomical theatre in Dublin, who had great opportunities of observing the post mortem appearances of fever. In the midst of all the discussions (says Mr. Kirby) relative to topical congestion, it was impossible not to remark the singular want of accordance between the prevalent opinions and the local appearances.

" The brain, so constantly supposed to be the seat of inflammation, *rarely* exhibited the characters indicative of such a state—in some instances, this organ was much paler than usual; in a very few, amongst a great number of dissections, was there any evidence of sanguineous or serous effusion; nor was the latter to any considerable extent. The *veins* on the surface of the brain often appeared in a state of unusual plenitude ;† I do not think, however, this circumstance affords any argument in favour of preceding inflammation; nor can we be certain to what degree this state of fullness existed before death. The contents of the thorax were

* See a considerable coincidence of opinion between Dr. Stoker and our correspondent Atticus.—Rev.

† See Atticus, in our last, page 446.

generally exempt from any character of recent disease, and rarely exhibited such marks as are esteemed to denote preceding inflammation."

The same might be said of the abdominal viscera.

"In short, (says Mr. Kirby) in a great majority of cases, so little did any particular organ seem to suffer, that I have wondered what could have been the cause of death." P. 432.

We think that this testimony might check the pertinacity with which the partizans of local inflammation in fever, adhere to their doctrines. We have long endeavoured to show that local inflammation in fever was a mere contingency, which might or might not take place, according to the violence of the disease or the local weakness of any particular organ. We every day see fresh proofs accumulating of the truth of this doctrine.

But to return. Dr. Stoker avers, that all the causes of typhus, whether predisposing or exciting, seem to be debilitating; contagion being probably the most so of all. When these causes are not excessive, he thinks, or when constitutional energy is not deficient, reaction is produced in the system equal to prevent or remove the morbid impression. Hence the large proportion of *spontaneous* recoveries. It is to the vigour of this reaction, he imagines, that we may attribute the extraordinary recoveries under the most opposite, and sometimes pernicious modes of treatment, which are employed or advocated by different authors—all of whom appeal to the recoveries of those submitted to their charge, as tests of their success.

"The injudicious or untimely employment of stimulants, or negligence in correcting those partial infarctions or congestions which arise from impeded *functions* in the early stages of fever, may change this reaction from a sanative process to a morbid excess, especially in the young, plethoric, or those of sanguine temperament; or, at certain seasons of the year, may endanger lesions in the vital organs, and hence inflammation—or indirectly may lead, by exhaustion, to increase the debility incident to the fever itself." P. 434.

This very view we have long ago given to the world, and we are happy in the coincidence of such men as Stoker and Atticus.

Dr. Stoker has very generally observed crises in fever, and those on critical days; indeed, he says, his subsequent experience has more and more tended to convince him that periodical movements occur almost as constantly in the regular continued fevers of this country, as in the exanthemata; the eruptions accompanying the latter rendering the periods in them more observable.

Our author traces the epidemic in Ireland to deficiency and vitiation of nutriment, with all the mental and corporeal miseries resulting from famine and poverty. He is not, however, prepared to deny that a certain atmospheric constitution might have had its share in the production of the fever—a cause of pestilence known to the ancients, and beautifully described by Virgil.

> ——— subito cum tabida membris
> Corrupto cœli tractu miserandaque venit
> Arboribusque satisque lues et lethifer annus
> Linquebant dulces animas aut ægra trahebant
> Corpora.

Without denying contagion, Dr. Stoker only contends that the contagion was not very active till the warmth of summer.

We shall not enter into the symptomatology of Dr. S. It is drawn up with great accuracy and fidelity, but does not differ, of course, in any material point, from that of Dr. Cheyne, or other physicians of the Irish metropolis.

In the treatment, as may be easily imagined from Dr. Stoker's views of the disease, he was rather disposed to select than multiply remedies. The following classification is, he thinks, pretty nearly in the order of utility. Cleanliness; ventilation; cool regimen; plentiful dilution; purgatives; antimonials; yeast; wine and camphorated mixture; emetics; cold or tepid ablution; blisters; partial fomentations.

Venæsection and mercury, he does not think generally necessary in the common fever of Ireland, unless in particular cases of plethora or biliary derangement. Dr. S. however, gives the following qualification of his anti-venæsectionary doctrines, promulgated a few years back in his work on fever.

> "Yet, I am free to acknowledge that moderate blood-letting *now* appears to me not to be so injurious in *complicated* cases as I *formerly* supposed, but certainly not more generally necessary in *simple* typhus." P. 462.

Finally, Dr. Stoker informs us, that *contagion* has been unusually active during the last two months of the time included in the report, a greater number of the hospital attendants having been infected than in any equal period previously.

In the same volume will be found, a very able Report of the epidemic fever of 1817-18, by Dr. F. Barker, of which we shall endeavour to give a brief analysis.

The topographical, climatorial, and economical observations in the first part of Dr. Barker's interesting paper, we must, for reasons already stated, pass over.

Dr. B. remarks, that the fever may be said to pervade all ranks of society in a nearly equal degree. Its chief *immediate* cause is, he thinks, contagion ; several striking proofs and examples of which he adduces. The *concurrent* causes he ascribes, with most others, to famine, unwholesome food, and the various depressing passions. Still these, he observes, can operate indirectly only, by rendering the human system more liable to receive the infection, and consequently by spreading it wider. Of the correctness of this position we entertain some doubts, since we believe, that all *predisposing* causes in one case may, when excessive in degree, become *exciting* or occasional causes in another.

A peculiarity of this epidemic, at its commencement chiefly, and during the winter season, was the occurrence of a livid or purple colour of the extremities, sometimes of the feet only, but generally of the hands also, not accompanied by coldness of those parts. Great disturbance of the sensorial functions mostly attended it. The symptom was formidable, but not mortal, as formerly observed to be. Increased determination to the brain, indicated by head-ache and delirium, was remarkable during the commencement of the epidemic—even local inflammations in external parts of the head were frequent.

During the first seven years, subsequent to the opening of the fever hospital, the mortality amounted to 1 in 12; but during the last seven years, it has scarcely amounted to 1 in 18. It is very easy to account for the different ratio of mortality in the Dublin hospitals, and in the London Fever Institution. Such is the extended accommodation for fevers in Dublin, that "patients now enter the hospitals at the rate of at least *two thousand monthly*;" and consequently, cases are received early in the disease. But in London, we are positive, when we assert, that nearly half the patients are brought in either moribund, or in so late a stage of the fever, as to preclude all attempts at cutting short or lessening the fatality of the disease.

In respect to the *post-mortem* appearances, Dr. Barker brings forward a testimonial from his friend Dr. Macartney, Professor of Anatomy, in Dublin. Dr. M. states that—

"Having reviewed his notes on the anatomical examination of persons who have died of typhus fever, he can state, as the result of his experience, that the morbid appearances in typhus fever are not those of common visceral inflammation. The morbid appearances that strictly belong to typhus are the following, according as the head, lungs, or abdominal viscera are engaged in the disease. 1st. Fullness or distention of the vessels of the brain, especially the veins—some water effused on the surface and into the cavities of the brain. 2d. The same species of congestion in the lungs, and different degrees of effusion in the cavities of the pericardium and pleura. 3d. Venous congestion in the liver, spleen, or alimentary canal, sometimes a blood-shot appearance or spots of extravasation in the mucous coat—more particularly in the stomach and first coils of the intestines. In some cases, a more generally pulpy or swollen and discoloured state of the mucous coat of the alimentary canal. These congestions were always of a purple or venous colour, and the blood throughout the body appeared to be accumulated in the venous system, and had little tendency to coagulate. Such were the appearances attendant on the congestions observable after typhus fever. The morbid appearances in real and pure inflammation are these: — 1st. In the head, the minute branches of the arteries appear more numerous than usual from carrying florid red blood. The effusion which takes place is more consistent than in the former case, and appears like whey; or pus is secreted on the membranes—the arachnoid coat is thickened and opake. 2d. In pleuritis and pericarditis, there is the same distribution of the arteries, and a wheyish-looking fluid, pus or lymph thrown out. In inflammation of the substance of the lungs there is always venous congestion, and the lungs feel more firm than in typhus.—3dly. In gastritis and enteritis the inflamed parts are denser, the redness is brighter than in typhus. The peritoneum is liable to be involved, and termination is slough or ulcer after a certain time. In cases where general fever is combined with real local inflammation, as sometimes occurs in dysentery, or when pneumonia is combined with typhus, or the latter with permanent and violent delirium, the peculiar morbid appearances of each disease are to be observed in combination.

"Two facts deserve to be recollected—1st. That the duration of general fever and visceral inflammation is not the same. 2d. That internal inflammations are very common in hot blooded animals, but idiopathic fever is peculiar to the human kind. It may be added, that processes of an inflammatory nature are fitted for repairing parts that have their functions interrupted or their structure injured, but the effects of typhus fever have no such power."

It is needless to say that we entirely coincide with Dr. Macartney in his valuable observations. The treatment pursued by our author, though simple, was far from Hippocratic. All patients, on their admission, were washed and cleansed, their clothes removed, and clean ones substituted.

"This is no unimportant part of the general treatment; it refreshes and produces a new state of the surface, with which the whole system so remarkably sympathises."

The hair was shaved off the head, with much benefit. The wards were kept clean and well aired; the patients freely supplied with drink; in cases of violent delirium, cold (frigorific) applications to the head were found useful; and when the determination to the head was great, arteriotomy or leeching was had recourse to, with a blister to the nape of the neck. When much local congestion was present, relief was obtained by general bloodletting, more especially if the symptoms indicated inflammation. Dr. B. disregarded the petechiæ, and adopted the principle of J. P. Frank, laid down nearly thirty years ago.

"In petechiis cum inflammatoria febre incedentibus, non posse modo sed debere sanguinem, pro impetus febrilis ratione educi jam diximus."

The cold affusion was sometimes employed with advantage; but patients were generally admitted at too advanced a stage for this remedy. The tepid affusion was more generally useful in many respects. In all cases, purgatives were exhibited to produce a sufficiently lax state of the bowels. In some desperate cases, where the patient's life appeared in imminent danger, in consequence of apparent congestion in the brain, small doses of calomel and antimonial powder, at short intervals of one or two hours, seemed to produce a favourable result. Wine is given in the hospital more sparingly than formerly, and the change is beneficial.

"Within the last two months, (says Dr. B.) I have witnessed the recovery of 150 patients, who did not receive on an average two ounces of wine."

The purple colour of the extremities already noticed, appeared to be diminished by detraction of blood from the head—"hence it would seem that this symptom arose from, or was connected with, congestion in the brain."

In the following passage we entirely coincide:

"No evil of great extent can exist without giving rise to some benefit, and it may result from the present epidemic, that witnessing many cases of recovery under very different modes of treatment, we shall cease to be dogmatically tenacious of system, and finding that induction, on a scale more extensive than hitherto deemed requisite, can alone furnish just conclusions, we shall gradually attain truth."

Upon the whole, we have been much gratified by the perusal of this document from the pen of Dr. Barker, though we cannot agree with him in his attempt to account for the origin of the present prevailing epidemic; namely, that "it has originated on the continent of Europe, and has been diffused by contagion, and extended to these countries in consequence of their constant and unusually great intercourse with the continent, at the time when contagious fever prevailed there."——

As an instance of the fallacy of facts and arguments brought forward by Dr. Barker, we may notice the following passage:

"The expedition to the island of Walcheren took place in the autumn of 1809, and it is stated by Sir G. Blane, in his Facts and Observations on the Walcheren Fever, that typhus was remarkably prevalent at Flushing. Consequently, among the 26,000 who sickened there, many must have laboured under infectious fever, and have conveyed it to these countries, to which they returned in great numbers." P. 582.

We can only say that we ourselves were at Walcheren from beginning to end of the expedition, that we almost every day visited the hospitals and sick quarters in Middleburg, Flushing, Camp Vere, and throughout the island of Beveland, without meeting these typhous or infectious fevers. And we confidently appeal to the profession at large, whether there is a single well authenticated instance of *any* of the Walcheren fevers being communicated to people in England.

Notwithstanding the length to which this article has extended, we cannot pass over the Medical Report of Dr. John O'Brien, without giving some account of so well constructed a paper. In tracing the moral and physical causes of this epidemic, Dr. O'Brien has feelingly touched on the *local* causes which, in Ireland, are unhappily laid deep in the frame of society, and arising from manners and habits generated by ages of civil and moral degradation, which has checked the natural progress of civilization, and perpetuated barbarism among that unhappy people,—"exhibiting a population increasing but not improving—blending most of the vices of civilization with the ignorance, apathy, sloth, and dirty habits of complete barbarism." P. 477.

Of the contagious nature of the present epidemic, Dr. O'Brien entertains no doubt; but he does not assent to the opinion that contagion is the *only* source of the disease. These fevers are, he thinks, capable of being also developed by different other poisons, either external, dependent on a vitiated state of the atmosphere; or internal, on a vitiated state of the animal fluids.

Dr. O'Brien has had, as yet, no reason for altering his opinions respecting the treatment of the fever, which were promulgated in the first volume of the Transactions; but he could not omit this opportunity of recording his dissent from the modern doctrine, that fever is to be considered one of the phlegmasiæ, and our treatment guided by this hypothesis.

"Although an inflammation, or sub-inflammation of different organs will frequently appear in the progress of this disease; yet, in my opinion, they are never to be considered as the primary disease, but as secondary symptoms, the *consequence* of unequal distribution of blood produced by the primary disease. With respect to the brain, which is the organ most seriously and frequently affected, it appears to me that of two very different conditions, its state approximates more to apoplexy from fullness of vessels, than phrenitis, the local vascular debility producing congestion or accumulation of blood in the vessels, of which coma and a species of delirium are the consequence. If you set about curing the delirious patient in typhus, as you would the phrenitic in phrenitis, you will find, in a great majority of cases, that death will be the consequence of those large detractions of blood which are indispensible in the latter disease." P. 490.

This, we are convinced, is sound pathology, and we really think that the advocates of the *phrenetic* theory would do well to cede a little to the general voice of the profession, as now unequivocally proved to be unfavourable to the doctrine—a doctrine which we think extremely dangerous in the medical community at large, inasmuch as there is every reason to fear that it may lead to an extravagant and injudicious use of venæsection, and ultimately bring it into disrepute—a fate which it has more than once undergone before, as the annals of medicine can testify.

From the foregoing analytico-synthetical sketch of the Medical Reports on the Fevers of Ireland, it will be evident to the reader, that a considerable coincidence of opinion and practice obtains among our brethren on the other side of the channel—a coincidence that is very pleasing to the philanthropic as well as the scientific mind. The few discrepancies of doctrine, or shades of variety in treatment, are less than could well be expected, under such circumstances, and are no more than are every day seen in a group of family likenesses.

——————— Facies non omnibus una,
Nec diversa tamen—qualis decet esse sororum.

Would that we may be able to say as much in our next, when we report on the fevers of this country!

